# Does human saliva decrease the antimicrobial activity of chlorhexidine against oral bacteria?

**DOI:** 10.1186/1756-0500-7-711

**Published:** 2014-10-10

**Authors:** Thaer Abouassi, Christian Hannig, Katja Mahncke, Lamprini Karygianni, Martin Wolkewitz, Elmar Hellwig, Ali Al-Ahmad

**Affiliations:** Department of Operative Dentistry and Periodontology, Albert Ludwigs University, Freiburg, Germany; Clinic of Operative Dentistry, Medical Faculty Carl Gustav Carus, TU Dresden, Dresden, Germany; Institute of Medical Biometry and Medical Informatics, Albert-Ludwigs-University, Freiburg, Germany

**Keywords:** Chlorhexidine (CHX), Saliva, Antimicrobial efficacy, Bacterial count

## Abstract

**Background:**

Several studies have shown the antibacterial effectiveness of 0.2% chlorhexidine (CHX) in both *in vitro* and *in vivo* studies. In this way, CHX comes directly in contact with saliva. This *in vitro* study aimed at investigating the possible neutralizing effect of saliva on CHX.

**Methods:**

Saliva samples (12 ml) were collected from twenty healthy volunteers. The aerobic and anaerobic bacterial counts in saliva were determined on Colombia blood agar (CBA) and yeast cysteine agar (HCB), respectively. Saliva from each subject was divided among 4 experimental groups (3 ml/group). Samples were centrifuged at 4000 g for 10 min. The centrifuged salivary bacteria were incubated with the following solutions: 0.2% CHX in saliva, CHX in saliva with 7% ethanol, CHX in 0.9% NaCl, CHX in 0.9% NaCl with 7% ethanol. After exposure for 1 min or 3 min to these CHX solutions, the CHX was neutralized and the bacteria were cultivated, after which the number of colony forming units (aerobic and anaerobic) was determined.

**Results:**

CHX reduced the CFU in all groups significantly (p = 0.0001). Therefore, CHX had a similar effect on both aerobic and anaerobic microorganisms. Significantly more bacteria survived the effect of CHX when kept in salivary solution. This effect from saliva could be compensated by the addition of ethanol. In the absence of saliva there was no significant difference observed in the effectiveness of CHX with respect to ethanol. Prolonging the exposure time to 3 min enhanced the effectiveness of CHX.

**Conclusions:**

The effect of saliva on the antimicrobial activity of CHX was weak albeit statistically significant. However, addition of 7% ethanol compensates this effect. The impact of saliva on the reduction of the antimicrobial efficacy of mouthrinses such as CHX needs to be taken into consideration with regard to improving their antibacterial properties.

## Background

Chlorhexidine digluconate (CHX) is a cationic biguanide which is bacteriostatic in low and bactericidal in higher concentrations [1]. It is more effective in alkaline than in acidic solution [2]. In addition to pH value, the antimicrobial activity of CHX is affected by the environment, i.e. by the presence of organic substances and food compounds, respectively, which reduce CHX activity [3]. CHX has demonstrated antibacterial activity on the salivary flora, as well as on the development of oral biofilms [4–6], but may be ineffective against biofilms which are already adherent or mature [7]. CHX at a concentration of 0.2% is regarded as the gold standard in the reduction of plaque formation and as a local antibacterial compound [8, 9]. Because of this, different forms of CHX are commonly used in dentistry. They can be found as varnishes in dental fissures to prolong CHX release in the oral cavity [8, 10], and as CHX-chips for local antibacterial therapy of periodontal pockets [11]. Furthermore, CHX as a gel, spray, or mouthwash has been described as an effective adjuvant to mechanical plaque control, especially during periodontal therapy [8, 12, 13]. Although the clinical use of CHX is well-documented, conflicting results indicate that its effectiveness might be influenced by a number of factors, such as application time in the oral cavity, mode of application and concentration [14]. Moreover, CHX comes in direct contact with the saliva which as a complex fluid contains numerous proteins and other ionic compounds able to bind to CHX, possibly resulting in its neutralization and subsequently in the selection of salivary bacteria. Portenier *et al.* demonstrated the inactivation of CHX by contact with the organic components of dentin and bovine serum albumin, respectively in an *in vitro* study [15]. However, in this study CHX diacetate and not digluconate was examined. CHX affects bacterial metabolic activity by interacting with the cellular membrane [16]. Due to its cationic nature, CHX binds to the phosphate groups in lipopolysaccharides (anionic compounds) in the bacterial cell wall, thereby interfering with membrane transport. As a result, leakage of low molecular weight molecules occurs [17]. Many studies have detected powerful antimicrobial action in the oral cavity, but there have been few investigations on the probable inactivation of CHX by the components of human saliva to date [3, 18].

The present study clearly draws attention to a probable inactivating effect of saliva on CHX. To investigate the efficacy of CHX *in vitro,* CHX neutralizers are necessary to stop the antimicrobial activity [19]. These neutralizers also contain complex mixtures of proteins such as the protease peptone [20, 21]. The working hypothesis of the present study was that human saliva, a fluid rich in proteins and ionic compounds, has a neutralizing effect on CHX, resulting in decreased reduction of aerobic and anaerobic salivary bacteria.

## Methods

### Subjects and samples

Stimulated human saliva (HS) from 20 volunteers was tested. The subjects were enrolled in the study only if the following criteria were met: no pregnancy, no drug treatment, no antibiotic therapy within the previous 3 months, and no use of mouth rinses two weeks before collection of saliva. The patients were between 23 and 49 years of age. Dental examination was carried out by an experienced dentist. There were no signs of open carious lesions or of periodontitis. The plaque index was close to zero. Stimulated saliva was collected by chewing paraffin 2 h after breakfast followed by brushing teeth with a particular commercial toothpaste product. Informed consent was taken from all volunteers to participate in this study.

### Study design and treatment of salivary bacteria

The pH of each salivary sample was measured using a pH meter (inoLab pH 720, WTW, Weilheim, Germany) and was in the range of 6.9-7.4. The aerobic and anaerobic bacterial load of the fresh saliva was determined as a baseline. Directly after collection saliva (12 ml) from each volunteer was divided among four experimental groups (3 ml/group). Samples were centrifuged at 4000 g for 10 min (Hettich, Tuttlingen, Germany). The resulting supernatants in group A and B were decanted into sterile cups and used to prepare 0.2% Chlorhexidine digluconate (CHX, Fagron, Barsbüttel, Germany) by diluting a 20% CHX stock solution. The supernatants in groups C and D were replaced by the same volume of saline solution.

Salivary bacteria obtained by centrifugation were treated in every group as follows: group A: 0.2% CHX in saliva supernatants without 7% alcohol, group B: 0.2% CHX in saliva supernatants with 7% ethanol, group C: 0.2% CHX in saline solution without 7% ethanol and group D: 0.2% CHX in saline solution with 7% ethanol. The antibacterial activity of CHX was evaluated after 1 min and 3 min. CHX was deactivated by adding CHX-neutralizer after 1 min or 3 min as described below.

### Neutralization of chlorhexidine digluconate (CHX)

The effectiveness of CHX after 1 min and 3 min was investigated using the CHX (Fagron, Barsbüttel, Germany) neutralizers as modified after Sheikh [21]. Briefly, there were two methods of neutralization used: Neutralizer No.1 consists of 3% asolectin (Fluka, Sigma-Aldrich Chemie GmbH, Steinheim, Germany), 10% Tween 80 (Tween® 80, Sigma-Aldrich Chemie GmbH, Steinheim, Germany), 0.3% sodium thiosulfate (Dr. Köhler Chemie GmbH, Alsbach-Hähnlein, Germany) in 0.1% aqueous proteose peptone (Becton Dickinson, New Jersey, USA). One ml of this neutralizer was added to 100 μl of the treated bacteria. Neutralizer No.2 is composed of 0.3% asolectin, 1% Tween 80, 0.3% sodium thiosulfate in 1% Tween 80. Neutralizer No. 2 (100 μl) was applied on the agar plates.

### Determination of the colony forming units (CFU)

The colony-forming units (CFUs) of both the collected saliva and of the different samples were evaluated quantitatively. For colony counting, dilutions (10^−1^-10^−5^) were prepared in 0.9% NaCl solution, 100 μl each of the different dilutions were plated onto yeast-cysteine blood agar (HCB) to cultivate anaerobic microorganisms at 37°C for 7 d (anaerobic jar, Anaerocult A; Merck, Darmstadt, Germany). Columbia blood agar (CBA) plates were used to cultivate aerobic and facultative anaerobic microorganisms after incubation at 37°C under a 5% CO_2_ atmosphere for 3 d.

### Statistical analysis

For aerobic and anaerobic cultivation a linear mixed model was fitted [22]. The response variable was the logarithm of the number of CFU. It was modelled as a linear function of time (1 min and 3 min), with group and the time-group interaction as explanatory variables. To take the dependency within each proband into account, the proband was fitted as a random effect. Variance components were used as a covariance structure. Model assumptions were evaluated graphically by residuals and other regression diagnostics including Cook's distance to indicate data points that are particularly worth checking for validity. Normality of the error terms was assumed. Least-square means were calculated with 95% CI and graphically displayed. Pairwise differences of least-square means were calculated and p-values were adjusted by the method of Tukey-Kramer to address the multiple test problem for pairwise comparisons [23]. All calculations were performed with the statistical software SAS system version 9.1.3 using the PROC MIXED procedure.

## Results

**Figure 1:**
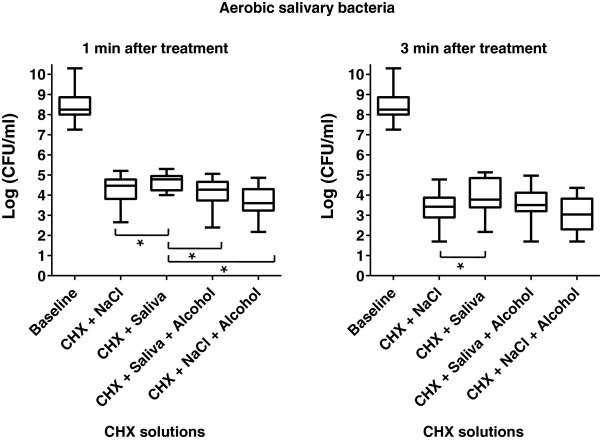
**Boxplots depicting bacterial count for surviving aerobic colony forming units (CFUs) determined after 1 min and 3 min treatments with 0.2% CHX digluconate in the different groups: centrifuged bacteria and 0.2% CHX in saliva without ethanol, centrifuged bacteria and 0.2% CHX in saliva with ethanol, centrifuged bacteria and 0.2% CHX in NaCl without ethanol, centrifuged bacteria and 0.2% CHX in NaCl with ethanol.** The medians and whiskers are displayed. The whiskers depict the range between minimum and maximum values. n = 20, *p ≤ 0.01 (Tukey-Kramer). The statistical significance regarding the bacterial counts was high (p ≤ 0.001) between each of the tested groups and the baseline.

**Figure 2:**
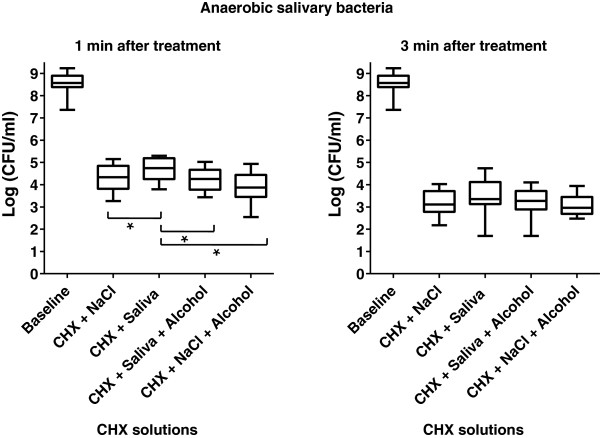
**Boxplots depicting the bacterial count of surviving anaerobic colony forming units (CFUs) determined after 1 min and 3 min treatments with 0.2% CHX digluconate in the different groups: centrifuged bacteria and 0.2% CHX in saliva without ethanol, centrifuged bacteria and 0.2% CHX in saliva with ethanol, centrifuged bacteria and 0.2% CHX in NaCl without ethanol, centrifuged bacteria and 0.2% CHX in NaCl with ethanol.** The medians and whiskers are displayed. The whiskers depict the range between minimum and maximum values. n = 20, *p ≤ 0.01 (Tukey-Kramer). The statistical significance regarding the bacterial counts was high (p ≤ 0.001) between each of the tested groups and the baseline.

The numbers of colony forming units (CFUs) in every group were calculated after 1 min and 3 min of treatment with 0.2% CHX, and statistically compared to the CFU number from untreated saliva (baseline). The results of aerobic and anaerobic CFUs grown after treatment of salivary bacteria are shown in Figures 1 and 2. In all groups the baseline bacterial count was reduced significantly by 0.2% CHX (p = 0.0001). No significant differences of CHX effects were observed when comparing aerobic and anaerobic bacteria. In comparison to the baseline bacterial load of fresh saliva, the aerobic CFUs were significantly less reduced after 1 min of action of CHX in salivary supernatant (group A, p = 0.0005, median 4.78). However, in this group the efficacy of CHX was significantly lower than that of CHX and 7% ethanol (group B, p = 0.0007, median 4.27), or CHX in 0.9% NaCl (group C, p = 0.009, median 4.47), or of CHX in 0.9% NaCl and 7% ethanol (group D, p = 0.0001, median 3.6). No statistical difference was found with regard to the effectiveness of CHX in groups B, C, and D. Furthermore, the anaerobic bacterial count was significantly reduced after 1 min of treatment as compared to the baseline (p = 0.0001). The four suspensions examined showed a comparable tendency for sheltering lower numbers of anaerobic microorganisms. Therefore, CHX in salivary supernatant (group A, p ≤ 0.001, median 4.74), CHX and 7% ethanol (group B, p ≤ 0.001, median 4.25), CHX in 0.9% NaCl (group C, p ≤ 0.001, median 4.33) and CHX in 0.9% NaCl and 7% ethanol (group D, p = 0.0001, median 3.87) presented significantly lower bacterial counts compared to the baseline.

Aerobic salivary bacteria were significantly reduced after 3 min of incubation in 0.2% CHX in all of the groups compared with the baseline level (median of baseline value 8.24, p = 0.0007). However, no significant difference in the effect of CHX was observed in saliva with or without 7% ethanol after 3 min. After 3 minutes, the effect of CHX in 0.9% NaCl solution (group C, p ≤ 0.001, median 3.42) was significantly more pronounced than in saliva (group A, p ≤ 0.001, median 3.78). There was no difference in the antibacterial effectiveness of CHX in groups C and D after 3 min (p ≥ 0.05).

With regard to anaerobic salivary bacteria, all of the tested suspensions allowed for a similar (p ≥ 0.05) level of microbial colonization after 3 min. However, the anaerobic CFU after 3 min of treatment with the above-mentioned suspensions were significantly reduced when compared to the baseline (median of baseline value 8.62, p = 0.0007).

## Discussion

The present study aimed to investigate possible neutralizing effects on chlorhexidine gluconate (CHX) by human saliva *in vitro*. Since CHX is a strongly cationic molecule it can react with anionic chemicals, resulting in inactivation of antimicrobial activity. The pH of unstimulated saliva ranges between 5.8 and 7.1, with an average daily flow between 1 and 1.5 l in healthy individuals [24, 25]. Portenier *et al*. [26] showed that the presence of dentine or bovine serum albumin caused a marked delay in the antibacterial effect of CHX. These results support the results of the present study. This may increase the resistance of some oral bacteria against its antibacterial action. Such presumptions were also made by Veksler *et al*. [27] with regard to deactivation of CHX by high organic load. The authors discussed the effect of pre-procedural rinsing with 0.12% CHX on aerobic oral bacterial strains during scaling and root planning procedures. They investigated possible inactivation of CHX by blood and debris arising from scaling and root planing, and found that CHX was not inactivated by this high organic load. In contrast, Lampe *et al*. [28] found that the antimicrobial activity of a gel containing 0.0156% CHX was decreased by human blood when tested against *Chlamydia trachomatis in vitro*. However, an influence of saliva on CHX with regard to bacterial reduction cannot be studied *in vivo*, since entry of bacteria from the tongue, mucosa and other parts of the oral cavity cannot be excluded. Therefore, an *in vitro* approach was chosen to study survival of oral bacteria after exposure to a clinically relevant concentration of CHX. For studies such as this it is important to stop the incubation of bacteria with CHX through effective deactivation of the residual antimicrobial activity of CHX. For this purpose a neutralising agent should be used which is itself not toxic to the tested bacteria. The protein-rich neutralizers which were used in this study have been reported as being non-toxic and effective in the deactivation of the antibacterial properties of CHX [20, 21]. The remaining cultivated bacteria can be considered as beingselected due to salivary components.

Herrera *et al.* [19] suggested resistance of some bacterial species such as *Peptostreptococcus micros*, *Capnocytophaga sputigena* and *Actinomyces naeslundii* against CHX. This resistance was shown in the absence of ethanol *in vitro*. The authors considered the absence of ethanol to be a reason for the observed resistance against CHX. Our results are in agreement with the study of Herrera *et al.* [19]. Furthermore, the present data indicated that saliva reduces the antibacterial activity of CHX and could be considered a reason for increased resistance of some oral bacteria against CHX. Many studies investigated the antibacterial effect of rinsing with CHX solutions shortly after periodontal treatment [29–31]. In these studies, CHX (0.2%) was investigated as the most frequently used concentration in dentistry. To date, the influence of CHX on salivary bacteria was studied in combination with testing of the antibacterial effects of CHX in the oral cavity [19, 32–34]. These studies aimed to investigate the effectiveness of different formulation of mouth rinses containing CHX. This issue is of practical relevance, but does not give information about the actual impact of human saliva on the antibacterial activity of CHX. However, entry of oral bacteria from parts of the oral cavity which were not in contact with CHX after rinsing could give false positive results with respect to resistance of oral bacteria in saliva. In the present study only salivary bacteria already exposed to the antibacterial activity of CHX in saliva or in physiological NaCl solution without ethanol or together with ethanol were isolated. This can give insight into the actual survival and resistance of salivary bacteria with or without the presence of human saliva, which can interact with CHX.

An inactivation of CHX by saliva has been investigated *in vitro* by Spijkervet *et al.* [3]. The authors dissolved CHX in human saliva and studied the minimum bactericidal concentrations (MICs) of CHX for different isolates of indigenous flora and for hospital-acquired microorganisms. The resulting MICs revealed a decreasing effect of human saliva on the antibacterial activity of CHX against hospital-acquired bacteria. The authors concluded that mouth rinsing with CHX is of limited value for decontamination of the oral cavity. However, in our study the total bacterial load of human saliva was exposed to CHX. There are no *in vitro* investigations on the influence of saliva on the antibacterial activity of CHX against highly diverse oral bacteria. This would not only detect a selection process for bacteria isolated from caries lesions, but might also give evidence about alteration of the physiological flora, which subsequently should be studied in more detail to identify which specific bacteria survive the effects of CHX in the presence and absence of human saliva.

The presence of ethanol in mouth rinse formulations is controversial. Some studies have hypothesized an increase in the risk of oral cancer [35]. However, some studies have suggested that the carcinogenic effect of ethanol only occurs if concentrations above 25% are used frequently [36]. Another study found no association of oral cancer with mouthwashes containing ethanol [37]. The present data have demonstrated a significant increase in antibacterial activity after mixing 0.2% CHX with 7% ethanol in saliva. This is in agreement with the suggestions of Herrera *et al.* [19]. This synergism could be caused by additional antimicrobial effects due to the 7% ethanol itself as well as its impact on salivary proteins. This could possibly compensate the neutralizing effects of these proteins.

Accordingly, human saliva can inactivate the antibacterial activity of CHX against some oral bacteria to some extent, inducing selective processes in the bacterial populations of human saliva. However, the effect of saliva on the antimicrobial activity of CHX was weak albeit statistically significant. These processes should be studied by cultivation and classification of selected bacteria *in vitro*. Furthermore, the well-known enhancing effect of ethanol on the antibacterial activity of CHX was confirmed.

## Conclusions

Human saliva has a neutralizing effect on chlorhexidine (CHX). The addition of 7% ethanol can compensate this effect.

### Consent

This study was approved by the Ethics Committee of the Albert Ludwigs-University Freiburg (222/08).

## Abbreviations

CHX: Chlorhexidine
CFUs: Colony forming units
HCB: Yeast-cysteine blood agar
CBA: Columbia blood agar.
